# Electrical impedance tomography to guide mechanical ventilation for asymmetrical lung injury: a case report

**DOI:** 10.3389/fmed.2025.1675679

**Published:** 2025-10-16

**Authors:** Albina Musaj, Louis Tronchet, Corstiaan den Uil, Dolf Weller

**Affiliations:** Department of Intensive Care, Maasstad Hospital, Rotterdam, Netherlands

**Keywords:** lung injury, ventilators, mechanical, electrical impedance tomography, respiratory insufficiency, case report

## Abstract

During last years, new methods of advanced ventilatory monitoring have been introduced to implement lung-protective ventilation. We present a unique case of a 66-year-old female admitted to the ICU with severe unilateral lung injury due to COVID-19 pneumococcal superinfection. By combining electrical impedance tomography (EIT) and esophageal pressure measurements, individualized positive end-expiratory pressure (PEEP) titration was performed. Initially, high PEEP levels (18 cmH_2_O) were optimal. Over the course of a week, reflecting dynamic lung recovery, PEEP levels could be reduced to 8 cmH_2_O followed by extubation. This case emphasizes the importance of personalized PEEP titration in managing asymmetric lung injury, highlighting how EIT-based monitoring can optimize alveolar recruitment while minimizing overdistension.

## Introduction

So-called “lung-protective ventilation” is key in critically ill patients undergoing mechanical ventilation. Fundamentally, this would mean low tidal volume, low plateau pressure and low driving pressure (1–3). However, in asymmetrical lung injury, sufficient PEEP should be administered to recruit the injured lung, while avoiding overdistension of the healthy lung (4–6). In order to do so, additional information can be obtained with electrical impedance tomography (EIT) and transpulmonary pressure measurements (4, 7–11). We discuss titrating PEEP for several subsequent days using these techniques in a patient with unilateral lung injury.

## Case presentation

We present the case of a 66-year-old Caucasian female patient with a history of untreated B-cell chronic lymphocytic leukemia (CLL), hypertension, and a COVID-19 infection 2 years ago. The patient had a body mass index (BMI) of 31.5, consistent with obesity. The patient was admitted to the Intensive Care Unit (ICU) with hypoxemic respiratory failure due to a pneumococcal pneumonia while having a positive nasopharyngeal swab polymerase chain reaction for SARS-CoV-2. Laboratory tests demonstrated an elevated C-reactive protein of 428 mg/L (normal range 0–10 mg/L) and elevated leukocyte count of 118.5 × 10^9^ (normal range 4.0–10.0 × 10^9^). Chest radiography showed consolidations in the right lung. The initial antibiotic treatment consisted of cefotaxime and ciprofloxacin. Following a negative Legionella antigen test, ciprofloxacin was stopped. Furthermore, the patient was treated with dexamethasone 6 milligrams per day and she received Tocilizumab. Due to suspected insufficient B-cell activity, the patient was also administered COVID-19 convalescent plasma.

**Figure 1:**
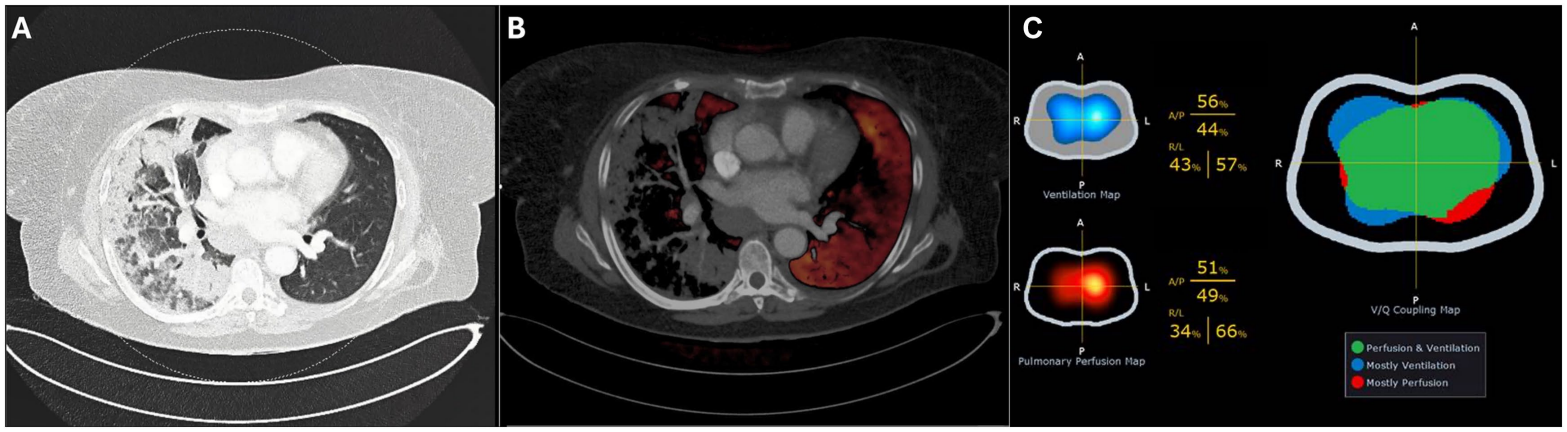
Dual-Energy CT in supine position showing the pulmonary window **(A)** and perfusion overlay **(B)**. Electrical impedance tomography (EIT) on day 4 **(C)** shows the ventilation and perfusion maps (left) and the ventilation–perfusion (V/Q) map (right), where matched regions appear in green, predominantly ventilated areas in blue, and predominantly perfused areas in red.

Upon admission to the ICU where high-flow nasal oxygen failed (flow 60 L/min, FiO_2_ 1.0, ROX index 2.68), the patient was sedated and intubated. Following intubation, a dual-energy computed tomography (DECT) scan of the chest in supine position revealed extensive consolidation with ground-glass opacities, predominantly affecting the right lung, with no evidence of pulmonary embolism. The DECT findings suggested significant hypoxemic vasoconstriction (Figure 1), predominantly in the right lung. Subsequently, the patient was placed in a prone position to address severe respiratory failure. The first EIT-guided positive end-expiratory pressure (PEEP) trial was then performed in prone position.

Simultaneous advanced respiratory monitoring was performed using esophageal pressure measurements (FluxMed, MBMed, Argentina), with correct catheter positioning confirmed by a Baydur occlusion test ratio of 1.00 and EIT (Enlight 1800/2100, Timpel Medical, São Paulo, Brazil). On days one, two, four, and five, EIT-guided decremental PEEP titration with steps of 2 cmH₂O every 30 s was performed. In our clinical practice, the optimal positive end-expiratory pressure was defined as the PEEP level at the step just before the crossing point, also known as the Costa method; this represents the PEEP setting that minimizes overdistension and collapse (12, 13). This usually results in a collapse below 5%, so we chose a PEEP level with a collapse below 5%. The initial PEEP titration in prone positioning on day 1 (from 20 to 6 cmH₂O) identified an optimal PEEP level of 18 cmH₂O, in prone position. Ventilation parameters over the course of the ICU admission are summarized in Table 1. The median trend and boxplots of PEEP settings and the P/F ratio from onset to ICU discharge are displayed in the Supplementary file.

**Table 1:** Ventilatory parameters during admission.

Mechanical ventilation parameters								
Variable	Day 0	Day 1	Day 2	Day 3	Day 4	Day 5	Day 6	Day 7
Mode^a^	HFNO/PC	PC	PC	PC	PC	PC/PS	PS	PS/HFNO
Positioning	Prone			Supine				
FiO_2_ (%)	100	40	40	50	35	35	35	35
P/F Ratio^b^	132	214	203	175	239	237	254	219
PEEP_e_ (cmH_2_O)	10	18	18	18	16	14	12	10–8
PC/PS level (cmH_2_O)	14	10	10	9	12	10	7	6
Respiratory Rate (/min)	24	28	28	28	28	28	21	15
I:E Ratio	1:1	1:1.5	1:1.5	1:1.5	1:1.5	1:1.5	na	na
VTe ml/kg/PBW^c^	na	6.2	6.3	6.8	7.3	7.7	7.8	7.6
Plateau Pressure (cmH_2_O)	na	26	27	25	26	21	na	na
Total PEEP (cmH_2_O)	na	19	19	19	19	15	na	na
Driving Pressure (cmH_2_O)	na	7	8	6	7	6	na	na
Compliance (ml/cmH_2_O)	na	46	50	64	59	67	67	97^d^
PEEP_i_ (cmH_2_O)	na	1	1	1	0	1	na	na
P_L,ei_ (cmH_2_O)	na	9.5	8.2	*na*	4.5	4.4	na	na
P_L,ee_ (cmH_2_O)	na	3.0	1.7	*na*	0.9	1.3	na	na
ΔP_L_ (cmH_2_O)	na	6.5	6.5	*na*	7.6	2.7	na	9^d^
ΔPes (cmH_2_O)	na	3.5	3.7	*na*	4.2	−4.8^e^	Pocc^f^	−2.8

a PC, Pressure Control (PC-CMV); PS, Pressure Support (PC-CSV); HFNO, High Flow Nasale Oxygen.

b Median.

c Mean expiratory tidal volume (24 h).

d Dynamic.

e Pes measurement during short PS period.

f Effort measurement: Pocc −8.3 cmH_2_O Pmus 6.23 cmH_2_O, Ptp 12.53 cmH_2_O.

After 24 h (day two), the patient was turned to the supine position, and a repeated PEEP titration (from 24 to 12 cmH_2_O) guided by EIT was performed on the second day, again identifying an optimal PEEP level of 18 cmH₂O. During this titration, a substantial degree of alveolar collapse of 38.5% at the lowest PEEP step and increased overdistension of 40.8% at the highest PEEP step were observed. Notably, after decreasing the PEEP from 24 to 18 cmH_2_O there was no collapse but from 18 to 16 cmH_2_O, a sudden increase in alveolar collapse from 0 to 29.6% was detected, coinciding with a decline in respiratory system compliance from 52.2 to 31.4 mL/cmH₂O. Over the following 24 h (day three), the patient remained ventilated with a PEEP of 18 cmH₂O. PEEP titration on day four identified an optimal PEEP of 16 cmH₂O. Additionally, perfusion imaging was conducted through EIT favoring the left lung with a distribution of 34%/66% (right/left) and ventilation distribution of 43%/57% (Figure 1C).

Over the course of the three measurements, a pattern indicative of lung recovery emerged. This was reflected in the changes in alveolar collapse at lowest PEEP level (28.6, 38.5, and 20%), a progressive increase in overdistension at the highest PEEP level (9.8, 40.8, and 45%), and an improvement in highest compliance (47.9, 52.2, and 70 mL/cmH₂O).

On the fifth day, the final EIT-guided PEEP titration was performed, revealing an optimal PEEP of 8 cmH₂O. The abrupt change in the previously set PEEP of 16 cmH₂O and the subsequent EIT-determined optimal PEEP of 8 cmH₂O led to a discussion within the ICU team. Based on P/F ratio, lung compliance, transpulmonary pressure monitoring (P_L,EE_), and arterial blood gasses, a stepwise PEEP reduction of 2 cmH₂O every 12 h was proposed.

On the same day, the patient successfully transitioned to pressure support ventilation. On day six, effort and transpulmonary pressure were monitored by measuring the ΔPocc (14). Due to the absence of available skilled ICU professionals to measure Delta Pes, airway occlusion pressure was measured as an alternative to assess effort. By day seven, the patient was receiving pressure support ventilation with a PEEP of 8 cmH₂O and a pressure support level of 6 cmH₂O. A spontaneous breathing trial (SBT) was performed, which the patient successfully passed. Following a brief period of continued ventilation, the patient was extubated and started on high-flow nasal oxygen therapy (flow 60 L/min, FiO₂ 0.5). Six days after extubation, the patient was discharged from the ICU.

**Figure 2:**
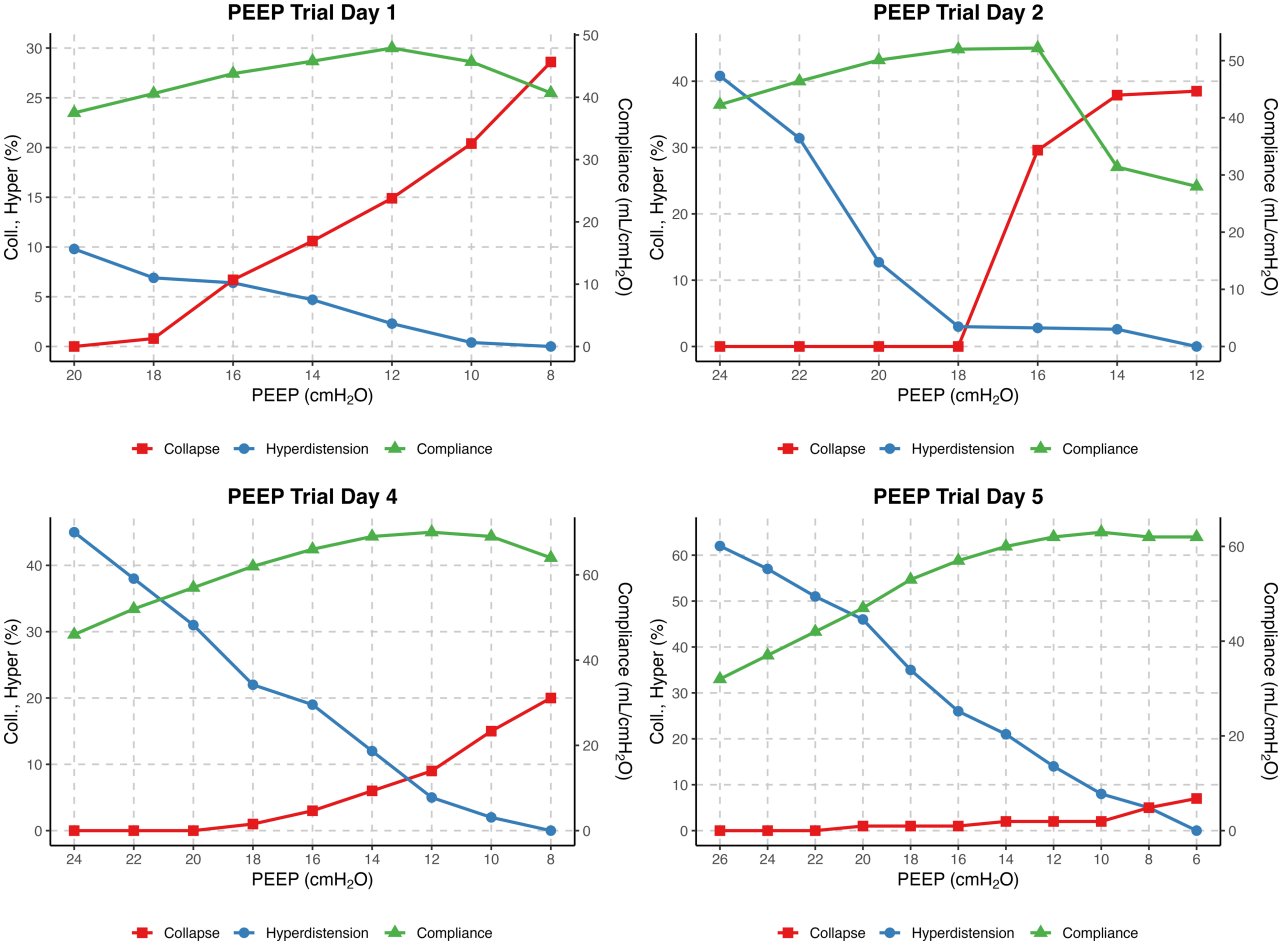
EIT-guided decremental PEEP titrations on Days 1, 2, 4 and 5. The x-axis shows PEEP (cmH₂O) from high (left) to low (right). The left y-axis shows collapse and overdistension (%), and the right y-axis shows compliance (mL/cmH₂O). Curves display collapse (red), overdistension (blue), and compliance (green) for each day.

The four EIT-guided PEEP titrations are graphically represented in Figure 2. The evolution of compliance, collapse and overdistension over time are illustrated in Supplementary Figure S2.

## Discussion

This case highlights the value of near-daily advanced pulmonary monitoring in a mechanically ventilated patient with severe unilateral lung injury. Serial EIT-based PEEP titrations, combined with esophageal pressure measurements, provided important insights into dynamic changes over time and supported individualized management strategies. Computed tomography remains the gold standard for assessing lung aeration (15), but it provides only a static image and involves risks during transport. EIT, in contrast, offers continuous, non-invasive, radiation-free bedside monitoring that can track changes in ventilation and the effects of therapy in real time.

Unilateral lung injury is common in mechanically ventilated patients, and several studies have explored ventilation strategies to optimize oxygenation while minimizing lung injury. Lateral positioning (“good lung down”) may enhance recruitment and improve gas exchange but also carries the risk of atelectasis in dependent regions (4–6, 16). Prone positioning promotes a more uniform distribution of ventilation by reducing vertical pleural pressure gradients, thereby limiting lung damage (17). These physiological effects explain why prone positioning equalizes regional ventilation and compliance (17–19). In our patient, the EIT-derived optimal PEEP was identical in prone and supine (18 cmH₂O), which may reflect persistently elevated pleural pressures associated with obesity (BMI 31.5). In the prone position, ventilation was more evenly distributed between dependent and nondependent lung regions, and this distribution appeared less dependent on PEEP than in the supine position (17, 20). The appearance of airway closing pressure in supine is consistent with less uniform pleural pressure distribution and greater dependent airway compression—reflecting increased chest wall weight and abdominal fat in the supine position (21, 22).

PEEP remains a key parameter in mechanical ventilation, but no consensus exists on the optimal strategy for unilateral injury. Individualized titration guided by EIT has shown promise in balancing recruitment against overdistension, providing real-time functional information at the bedside (8, 23–25). Sousa et al. reported that individualized PEEP improved pulmonary hemodynamics and function, especially in bilateral injury, whereas in asymmetric cases optimal PEEP values varied less, but higher PEEP increased overdistension of the non-injured lung (6, 26).

Esophageal pressure (Pes) measurements have also been proposed as a complementary tool. Bastia et al. (4) showed that maintaining an end-expiratory transpulmonary pressure (P_L,EE_) of 0 cmH₂O promotes a homogeneous distribution of tidal volumes, preventing collapse in the injured lung and hyperinflation in the non-injured lung. In contrast, our findings differed: P_L,EE_ ranged from 0.9 to 3.0 cmH₂O (median 1.5) when PEEP was selected according to the <5% collapse strategy. A likely explanation is that this approach resulted in choosing a PEEP level close to the crossing point, or that the PEEP steps used during titration — particularly the highest and lowest levels applied — influenced the degree of collapse and overdistension (25). Evidence directly comparing EIT- and Pes-guided strategies for individualized PEEP remains limited.

We follow a standardized operating procedure (SOP) for PEEP titration, commencing at 10 cmH₂O above the previous setting, with a minimum of 24 cmH₂O, followed by a decremental trial in 6–8 steps until reduced compliance, oxygen desaturation to <90%, or collapse >10% was observed (to prevent derecruitment). In our patient, the protocol was limited to 20 cmH₂O on day one due to hemodynamic deterioration (ABP 72/58 mmHg, MAP 63 mmHg, desaturation to 79%). On day two, the PEEP trial was performed from 24 cmH₂O down to 12 cmH₂O (Figure 2). We acknowledge that the restricted range of PEEP values may have influenced the EIT-derived optimal PEEP, as algorithms assume that overdistension is 0% at the lowest PEEP and collapse is 0% at the highest step (8). Consequently, the relatively high lowest PEEP level may have underestimated overdistension and shifted the calculated crossing point toward a higher PEEP. This limitation underlines the importance of standardized EIT protocols, as recently emphasized by Scaramuzzo et al. (27). After the 18 cmH₂O PEEP step, collapse suddenly increased from 0 to 30% and reached 40% at 12 cmH₂O. According to our SOP, this high degree of collapse was the reason to terminate the trial at 12 cmH₂O. Based on the shape of the curve, this point most likely represents the airway closing pressure. EIT-guided PEEP titration was not performed on day 3 due to lack of experienced personnel, which may have reduced insight into disease progression. As shown in Supplementary Figure S1, the P/F ratio decreased between days two and three, with improvement after titration on day four. In retrospect, daily PEEP trials would have been preferable. Perfusion–ventilation mismatch measurements were performed only on day 4 (Figure 1C); although consistent with DECT results, conclusions remain limited given the single measurement.

In addition, the patient’s untreated chronic lymphocytic leukemia (CLL) likely contributed to impaired immune responses and increased susceptibility to secondary infection. CLL-associated immunosuppression may have aggravated the severity of the pneumococcal pneumonia and influenced the heterogeneity of lung injury, which could have affected both disease course and ventilatory management.

Barriers to the clinical implementation of EIT include limited availability and the costs of devices and disposables. Nevertheless, potential benefits include reducing the need for transport CT scans and preventing ventilator-induced lung injury. Lung ultrasound (LUS) represents a widely available alternative but also has limitations compared to EIT such as operator-dependency and anatomical limitations. Ongoing research will further clarify the clinical value and broader applicability of EIT in patients with acute lung injury.

## Conclusion

This case report describes a patient with severe unilateral lung injury resulting in profound oxygenation impairment. During the first 5 days of intensive care management, advanced monitoring techniques were employed, including PEEP titrations guided by electrical impedance tomography (EIT) and esophageal pressure measurements. Significant fluctuations in lung compliance and alveolar collapse were observed, with optimal PEEP levels ranging between 18 cmH₂O and 8 cmH₂O over the course of treatment. This case highlights the critical role of individualized PEEP titration in adapting to the dynamic changes in lung mechanics in patients intubated for asymmetrical lung injury. Based on this case, daily PEEP titration with advanced monitoring is recommended to optimize PEEP settings, adapt to changes in lung mechanics, and minimize both alveolar collapse and overdistension.

## Data Availability

The original contributions presented in the study are included in the article/Supplementary material, further inquiries can be directed to the corresponding author.

## Author’s note

Analyses and plots were generated using R version 4.5.1 (June 2025).
