# Supplementary figures and images for: Electrical impedance tomography to guide mechanical ventilation for asymmetrical lung injury: a case report
Source: Front Med (Lausanne). 2025 Oct 16;12:1675679. doi: 10.3389/fmed.2025.1675679 (PMC12571728; doi:10.3389/fmed.2025.1675679)

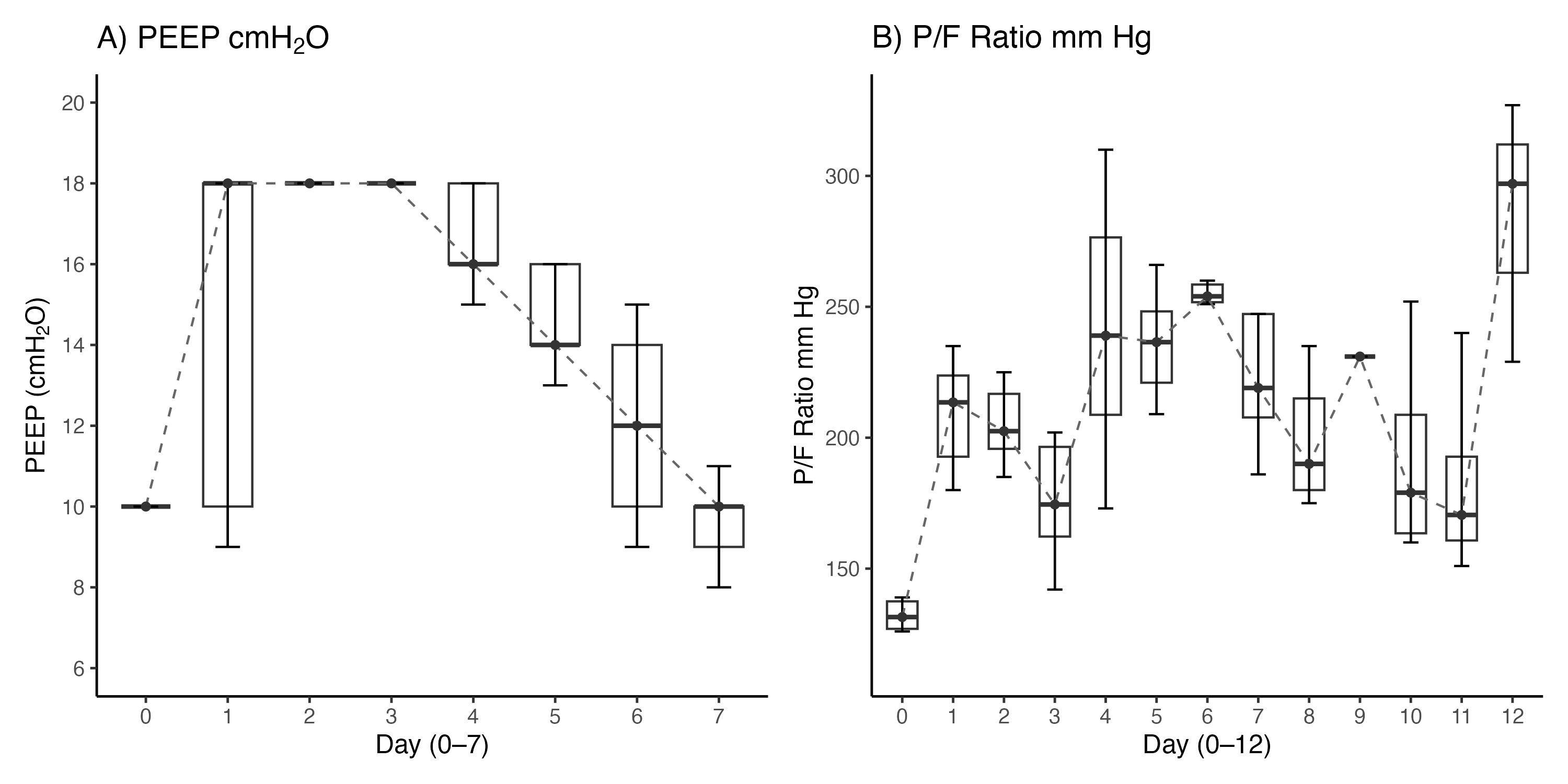

Supplement: Supplementary file 1 [file Image_1.tiff]

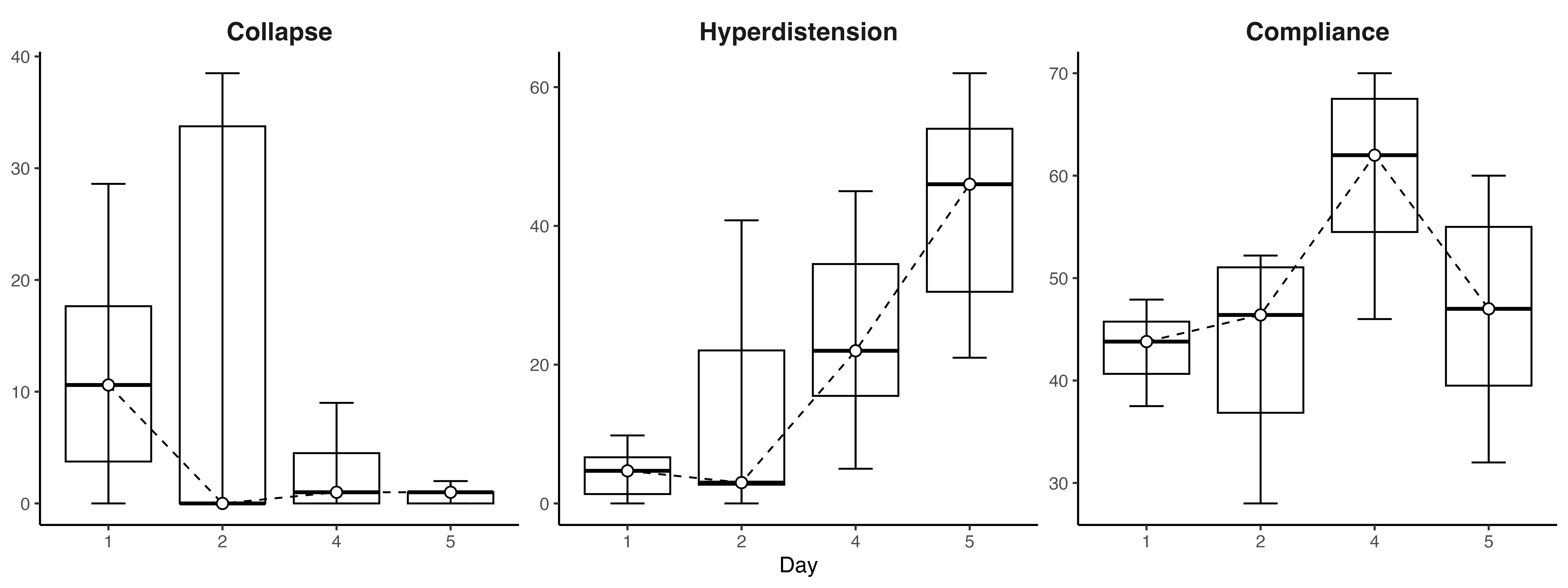

Supplement: Supplementary file 2 [file Image_2.tiff]
